# Active monitoring of antifungal adverse events in hospitalized patients based on Global Trigger Tool method

**DOI:** 10.3389/fphar.2024.1322587

**Published:** 2024-06-28

**Authors:** Xiao Meng, Yaozhou Wu, Zixuan Liu, Yifan Chen, Zhizhou Dou, Li Wei

**Affiliations:** Department of Pharmacy, The First Affiliated Hospital of Guangzhou Medical University, Guangzhou, Guangdong, China

**Keywords:** Global Trigger Tool, adverse drug events, antifungal drugs, retrospective study, adverse (side) effects

## Abstract

**Background:**

The increasing prevalence of fungal infections necessitates broader use of antifungal medications. However, the prevalence of adverse drug events (ADEs) restricts their clinical application. This study aimed to develop a reliable ADEs trigger for antifungals to enable proactive ADEs monitoring, serving as a reference for ADEs prevention and control.

**Methods:**

This investigation comprises two phases. Initially, the trigger was established via a literature review, extraction of relevant items, and refinement through Delphi expert consultation. Subsequently, the validity of the trigger was assessed by analyzing hospital records of antifungal drug users from 1 January 2019 to 31 December 2020. The correlation between each trigger signal and ADEs occurrence was examined, and the sensitivity and specificity of the trigger were evaluated through the spontaneous reporting system (SRS) and Global Trigger Tool (GTT). Additionally, risk factors contributing to adverse drug events (ADEs) resulting from antifungal use were analyzed. Results: Twenty-one preliminary triggers were refined into 21 final triggers after one expert round. In the retrospective analysis, the positive trigger rate was 65.83%, with a positive predictive value (PPV) of 28.75%. The incidence of ADEs in inpatients was 28.75%, equating to 44.58 ADEs per 100 admissions and 33.04 ADEs per 1,000 patient days. Predominant ADEs categories included metabolic disturbances, gastrointestinal damage, and skin rashes. ADEs severity was classified into 36 cases at grade 1, 160 at grade 2, and 18 at grade 3. The likelihood of ADEs increased with longer stays, more positive triggers, and greater comorbidity counts.

**Conclusion:**

This study underscores the effectiveness of the GTT in enhancing ADEs detection during antifungal medication use, thereby confirming its value as a monitoring tool.

## 1 Introduction

Adverse drug events (ADEs) refer to any harm resulting from the use of medication, including physical, psychological, or functional impairments (Bates et al., 1995). ADEs account for approximately 19% of adverse events in hospitalized patients (Leape et al., 1991), and globally, they lead to 770,000 patient disabilities or deaths each year, affecting 3.6% of all admitted patients (Tatonetti et al., 2012). In China, the number of adverse drug reactions/event reports has been steadily increasing since 1999. The most recent national annual report on adverse drug reactions/events revealed a total of 1.962 million reports received in 2021. Among them, 597,000 reports were new and severe, accounting for 30.4% of the total reports during the same period. Additionally, at present, all Adverse Drug Events (ADEs) in medical institutions in our country are reported voluntarily by healthcare professionals, including medical, nursing, and pharmacy staff. This method suffers from issues such as inaccurate, incomplete, and outdated information, varying levels of awareness and understanding among reporters, high bias in reporting, difficulty in identifying rare and special ADEs, and a high rate of underreporting. A significant number of ADEs are not reported, making it impossible to accurately determine the incidence rate of ADEs in medical institutions.

The Global Trigger Tool (GTT) was introduced in 2003 by the American Institute for Health Promotion as an active monitoring tool for ADEs. This retrospective method involves reviewing the original medical records of hospitalized patients and utilizing specific “triggers” to identify potential ADEs. In comparison to the traditional voluntary reporting approach, the GTT method offers notable advantages in ADEs detection. However, when applying GTT in different regions and study populations, it is essential to enhance the triggers based on its corresponding clinical medication characteristics, demographic characteristics, social characteristics, and cultural characteristics (Björnsson and Olsson, 2006; de Souza et al., 2016; Haukland et al., 2017; Chai et al., 2022; Mishra et al., 2022).

Existing research on GTT is primarily based on population studies, with almost no drug-specific research. Due to the many confounding factors in population studies, there is a certain lack of specificity and sensitivity in the triggers. Triggers designed for specific drugs may yield better results. Taking into account the actual situation of our hospital, in recent years, the increasing number of patients with malignant tumors, severe infections, and AIDS, as well as the widespread use of immunosuppressants, broad-spectrum antibiotics, and glucocorticoids, has led to a growing number of patients with fungal infections. Consequently, the clinical application of antifungal drugs is becoming more extensive. However, most antifungal drugs lack specificity and can be toxic to host cells but can target fungi, making ADEs a significant limitation in their clinical use (Kim, 2016). Therefore, it is vital to set up a trigger for monitoring antifungal-induced ADEs to promptly identify them and promote rational drug use in clinical care. However, despite an extensive literature review, no studies on the application of GTT for monitoring ADEs caused by antifungal drugs have been reported to date. Therefore, this study aimed to establish a trigger for antifungal drugs and conduct a retrospective study on its clinical applicability, providing a reference for the active detection and prevention of ADEs in the future use of antifungal drugs.

## 2 Methods

### 2.1 Trigger establishment

#### 2.1.1 Literature search

We systematically searched the PubMed, MEDLINE, and CNKI databases for relevant literature from January 2000 to May 2022 using specific keywords such as Global Trigger Tool, Trigger Tool, Adverse Drug Reaction, Adverse Drug Events, and Antifungal. Our inclusion criteria were as follows: 1) studies with specific triggers; 2) studies focusing on hospitalized adult patients; and 3) studies involving the introduction of trigger applications and the development of new triggers. Despite a thorough literature search, no GTT studies applied to antifungal drugs were found as of May 2022.

Conduct a comprehensive statistical analysis and evaluation of the population, sample size, trigger tools, total detection rate, and positive predictive value (PPV) across the included literature. In the synthesis of the trigger tools used in the literature, arrange the frequency of each trigger tool item in descending order. Where items share the same frequency but differ in their thresholds, prioritize the one with the higher PPV as the threshold value for that particular trigger tool. The normal range, critical values, and interpretation of these trigger tools are provided for reference purposes.

#### 2.1.2 Trigger item extraction

Preliminary trigger items were extracted from the included literature. Afterward, considering the instructions for the use of antifungal drugs, relevant ADEs databases, and reference books, the trigger items were further refined around four modules: “laboratory indicators, clinical symptoms, antidotes, and intervention measures.” The extracted results were thoroughly evaluated by a review team consisting of a pharmacist and a physician.

#### 2.1.3 Delphi expert consultation method to optimize trigger items

Based on the work presented in Section 2.2, we utilized the Delphi method (Olsen et al., 2021) to conduct expert surveys. A total of 15 senior clinical medical and pharmaceutical experts who were actively involved in frontline anti-infection work were randomly selected from eight different tertiary medical institutions in China for questionnaire consultation. The initial set of triggers was revised based on expert suggestions, including the basic principles and explanations of the input parameters. After one round of revision, triggers that showed high consistency among experts were retained, resulting in the development of a trigger that was highly recognized by the experts.

### 2.2 Trigger validation

According to domestic and foreign literature reports, the incidence rate of ADEs in hospitalized adult patients varies from 3% to 16% (Silva et al., 2019). For this study, a *P* value of 10% was selected, and using the formula *N* = Z^2^×P×(1−P)/δ^2^, with a 95% confidence level (Z = 1.96) and a 3% sampling error (δ), the sample size N was calculated to be 384. To ensure adequate representation, a random sampling method was used to select 480 patients who had used antifungal drugs in the hospital between 1 January 2019, and 31 December 2020. Cases were drawn monthly from the Hospital Information System, with 20 cases selected each month. The inclusion criteria for these patients were as follows: 1) systemic administration (oral, intravenous injection) of itraconazole, fluconazole, voriconazole, posaconazole, caspofungin, and amphotericin B for ≥24 h; 2) patient age ≥28 days; and 3) hospital stay ≥24 h.

This study received approval from the Ethics Review Committee of the First Affiliated Hospital of Guangzhou Medical University (No. ES-2023-178-01).

### 2.3 Retrospective record review

A team comprising two junior reviewers and two senior reviewers were assembled to conduct the record review. Prior to the case review, all team members underwent ADEs training. The medical records were independently reviewed by the two junior reviewers to determine whether the trigger result was positive or false positive. The review included several aspects, including the first page of the medical records, disease progression, medication orders, laboratory tests, imaging examinations, nursing records, and admission and discharge records. In cases where both junior reviewers identified the trigger as positive, a causality assessment of the suspected drug occurred, resulting in the inclusion of the case for further study. Any disagreements were resolved by the two senior reviewers, who made the final determination. Additionally, during the review process, patient information, such as sex, age, length of hospital stay, medication allergy history, number of medications, trigger-specific information, and occurrence time, was recorded. Each patient’s medical record was allocated 20 min for review, adhering to the “20-min rule” for all records regardless of size.

The causality assessment of ADEs was conducted in accordance with the World Health Organization-Uppsala Monitoring Centre (WHO-UMC) criteria, which consists of six levels: certain, probable, possible, unrelated, unclassifiable, and unassessable (Table 1) (Sendekie et al., 2023). The severity of ADEs was determined using the Common Terminology Criteria for Adverse Events (CTCAE) version 5.0, which provides specific clinical descriptions for severity levels 1 to 5 (Table 2) (Hu et al., 2022).

**TABLE 1:** WHO-UMC causality categories.

Causality term	Assessment criteria
Certain	• Event or laboratory test abnormality, with plausible time relationship to drug intake
	• Cannot be explained by disease or other drugs
	• Response to withdrawal plausible (pharmacologically, pathologically)
	• Event definitive pharmacologically or phenomenologically (i.e., an objective and specific medical disorder or a recognized pharmacological phenomenon)
	• Rechallenge satisfactory, if necessary
Probable/Likely	• Event or laboratory test abnormality, with reasonable time relationship to drug intake
	• Unlikely to be attributed to disease or other drugs
	• Response to withdrawal clinically reasonable
	• Rechallenge not required
Possible	• Event or laboratory test abnormality, with reasonable time relationship to drug intake
	• Could also be explained by disease or other drugs
	• information on drug withdrawal may be lacking or unclear
Unlikely	• Event or laboratory test abnormality, with a time to drug intake that makes a relationship improbable (but not impossible)
	• Disease or other drugs provide plausible explanations
Conditional/Unclassified	• Event or laboratory test abnormality
	• More data for proper assessment needed, or additional data under examination
Unassessable/Unclassifiable	• Report suggesting an adverse reaction
	• Cannot be judged because information is insufficient or contradictory
	• Data cannot be supplemented or verified

**TABLE 2:** CTCAE5.0 general guideline.

Grade 1	Mild; asymptomatic or mild symptoms; clinical or diagnostic observations only; intervention not indicated
Grade 2	Moderate; minimal, local or noninvasive intervention indicated; limiting age-appropriate instrumental ADL^a^
Grade 3	Severe or medically significant but not immediately life-threatening; hospitalization or prolongation of hospitalization indicated; Disabling; limiting self-care ADL^b^
Grade 4	Life-threatening consequences; urgent intervention indicated
Grade 5	Death related to AE

ADL is short for Activities of Daily Living.

^a^ Instrumental ADL, refers to preparing meals, shopping for groceries or clothes, using the telephone, managing money, etc.

^b^ Self-care ADL, refers to bathing, dressing and undressing, feeding self, using the toilet, taking medications, and not bedridden.

### 2.4 Statistical analysis

Data analysis was performed using Microsoft Excel 2019 and SPSS 24.0 software. The rank sum test and chi-square test were utilized to compare quantitative and qualitative data, the application of sensitivity, specificity, positive predictive value (PPV), accuracy index, and Kappa value to evaluate the GTT method, whereas the binary logistic regression method was employed to analyze the influencing factors of ADEs associated with antifungal drugs. ADEs per 1,000 patient days, ADEs per 100 admissions, and the occurrence rate of ADEs in hospitalized patients were calculated. The ADEs per 1,000 patient days served as an indicator to track the occurrence of ADEs over time.

## 3 Results

### 3.1 Trigger establishment

Based on our inclusion criteria, we included a total of 23 articles (Franklin et al., 2010; Kaafarani et al., 2010; Classen et al., 2011; Carnevali et al., 2013; Rozenfeld et al., 2013; Hwang et al., 2014; Guzmán-Ruiz et al., 2015; Härkänen et al., 2015; Karpov et al., 2016; de Almeida et al., 2017; Bhise et al., 2018; Silva et al., 2018; Zimlichman et al., 2018; Grossmann et al., 2019; Hu et al., 2020; Schulson et al., 2020; Xu et al., 2020; El et al., 2021; Hwang et al., 2021; Otero et al., 2021; Toscano et al., 2021; Gohil et al., 2022; Valkonen et al., 2023), most of which explored triggers mentioned in the GTT white paper. A total of 21 trigger factors were identified from various sources. After one round of expert survey, we refined the aforementioned factors and ultimately determined 21 triggering factors. These factors are primarily divided into four different modules, which consist of 7 laboratory test indicators (L), 10 clinical symptoms (S), 2 antidotes (A), and 2 clinical intervention measures (T).

### 3.2 Patient characteristics

In this study, we reviewed the medical records of 480 patients who were included, mainly from departments such as respiratory medicine, thoracic surgery, geriatrics, and neurology. The average age of the patients was 56.05 ± 19.77 years, with males accounting for more than half (66.7%). The average length of hospital stay was 13.49 ± 12.83 days, ranging from 1 to 112 days. The patients used an average of 1.22 ± 0.77 types of antifungal drugs in combination. The patients had an average of 5.41 ± 2.96 comorbid diseases. There was a statistically significant difference (*p* < 0.05) between patients with and without ADEs in terms of hospital stay, the number of antifungal drugs used in combination, the number of other antibiotics used in combination, the number of positive triggers, and the number of comorbid diseases (Table 3).

**TABLE 3:** Characteristics of patients with ADEs and with no ADE.

Characteristics	Patients with ADE (*n* = 338)	Patients without ADE (*n* = 142)	P
Age(years)			**0.175**
0–17	22	6	
18–40	51	18	
41–60	116	40	
≥61	149	78	
Gender			**0.12**
Male	218	102	
Female	120	40	
Department			**0.902**
Internal	273	114	
Surgery	65	28	
Number of admission(days)			<0.001*
1–10	215	48	
11–20	84	51	
21–30	16	20	
≥31	23	23	
BMI/kg/m^2^			**0.194**
≥18.5	77	34	
18.5–23.9	195	90	
≥24.0	66	18	
Number of previous hospitalizations			**0.842**
0–3	266	114	
4–6	35	15	
≥7	37	13	
Number of combined antifungal agents			<0.001*
1	296	106	
2	39	27	
3	3	9	
Number of combined antibacterials			<0.001*
0–3	302	101	
4–6	32	36	
≥7	4	5	
Number of positive triggers			<0.001*
0–2	298	62	
3–5	36	61	
≥6	4	19	
Number of combined diseases			<0.001*
1–3	117	22	
4–6	123	72	
≥6	98	48	

BMI, Body mass index.

Bolded and asterisked results indicate that the data is less than 0.05, which is statistically significant.

### 3.3 Trigger detection

Using the above 21 trigger factors, a comprehensive examination was conducted on 480 medical records. All 21 trigger factors yielded positive results in actual application. Among the 480 patients, 316 had positive triggers, resulting in a total of 795 trigger instances, averaging approximately 1.66 instances per patient. A total of 214 instances of ADEs caused by antifungal drugs were identified, involving 138 patients, with a PPV of 26.92% for the trigger. The occurrence rate of inpatients with ADEs was 28.75%. The incidence of ADEs per 100 admissions was 44.58, and that per 1,000 patient days was 33.04. The detailed results of the trigger monitoring are presented in Table 4.

**TABLE 4:** 21 triggers and positive predictive values for active monitoring of antifungal ADEs.

No.	Trigger	Positive triggers	ADEs	PPV (%)
L1	PLT < 50 × 10^9^/L	30	4	13.33
L2	WBC < 3.0 × 10^9^/L	40	11	27.5
L3	NE < 1.5 × 10^9^/L	38	10	26.32
L4	Rising BUN or serum creatinine greater than 2 times baseline/Creatinine clearance <50 mL/min	85	18	21.76
L5	ALT>84U/L or AST>80U/L (or ALP>121U/L and T-BIL>2UNL)	35	13	37.14
L6	K^+^<2.9 mmol/L	53	21	39.62
L7	Voriconazole >5 μg/mL	48	18	37.5
S1	Liver damage, hepatotoxicity, drug-induced hepatitis, liver injury, liver failure, liver function abnormalities, bilirubinemia, jaundice, cholestasis, yellowing of the skin and sclera, strong tea-like urine, potter’s clay-like stools, stuffy epigastric area	25	6	24.0
S2	Myalgia, bone pain, back pain, muscle pain, joint pain, periostitis	12	2	16.67
S3	Vision, optical impairment, photophobia, green eyesight, visual field defect, yellow eyesight, blue eyesight, partially sighted	51	8	15.69
S4	Rash, itching, erythema, hyperpigmentation, phototoxicity, photosensitivity reaction, drug rash, skin lesions	66	14	21.21
S5	Mental disorders, hallucinations, delirium, visual hallucinations, auditory hallucinations, hyperactivity, agitation, verbosity, depression, irritability, sensory abnormalities, somnolence, babbling, photophobia, psychotic symptoms	43	7	16.28
S6	Gastrointestinal discomfort such as nausea and vomiting	55	23	41.82
S7	Acute renal injury, sunken edema, general edema, oliguria, anuria	14	5	35.71
S8	Bleeding (including nosebleed, gingival bleeding, gastrointestinal bleeding, vaginal bleeding)	9	1	11.11
S9	Hypokalemia and myasthenia	53	17	32.08
S10	Phlebitis, infusion reaction, vascular redness and swelling	1	1	100
A1	Use of diphenhydramine/Loratadine/Chlorphenamine maleate/Cetirizine/Promethazine	71	18	25.0
A2	Use of live intestinal bacteria preparations/Antidiarrheal agents such as montmorillonite	56	15	26.79
T1	Abrupt cessation of medication	7	2	28.57
T2	Admission to ICU/Rescue	3	0	0
Total	—	795	214	26.92

PLT, platelet count; WBC, white blood cell; NE, neutrophilic granulocyte; BUN, blood urea nitrogen; ALT, alanine aminotransferase; AST, aspartate aminotransferase; ALP, alkaline phosphatase; T-BIL, total bilirubin; UNL, upper limit of normal; ADEs, adverse drug events; PPV, positive predictive value; ICU, intensive care unit.

Furthermore, during the period from 1 January 2019 to 31 December 2020, the hospital recorded a total of 4,282 patients who used antifungal drugs, with 35 spontaneous reports of ADEs involving 33 patients, resulting in a detected incidence rate of ADEs of 0.77%. The detection rate of ADEs associated with antifungal drug triggers in this study was significantly greater (*p* < 0.05) than that associated with spontaneous reporting.

### 3.4 Characteristics of ADEs

The identified ADEs can be categorized into nine main categories, with the highest number of cases affecting the metabolic and nutritional system (60 cases, 28.04%), followed by the gastrointestinal system (38 cases, 17.76%), the skin and appendages system (32 cases, 14.95%), and the hematopoietic and lymphatic system (25 cases, 11.68%) (Table 5). Based on the WHO-UMC assessment method, 193 cases of ADEs were considered possibly related (90.19%, 193/214), whereas 21 cases were classified as probably related (9.81%, 21/214). According to the CTCAE 5.0 grading system, 36 patients with ADEs were classified as grade 1 (16.82%, 36/214), 160 patients were classified as grade 2 (74.77%, 160/214), and 18 patients were classified as grade 3 (8.41%, 18/214). No cases of grade 4 or 5 severity were observed.

**TABLE 5:** ADEs involved system-organ classification.

ADE involves organs and systems	The occurrence of ADE	Percentage (%)
Metabolic and nutritional disorders (hypokalemia, high blood drug concentration)	60	28.04
Gastro-intestinal system disorders (abdominal pain, diarrhea, abdominal distension)	38	17.76
Skin and appendages disorders (rash, erythema, pruritus)	32	14.95
Blood and lymphatic system damage (white blood cell, neutrophils, platelets)	25	11.68
Urinary system damage (kidney injury, creatinine decrease)	23	10.75
Liver and biliary system disorders (elevated transaminase, hepatotoxicity)	19	8.88
Vision disorders (blurred vision)	8	3.74
Central and peripheral nervous system disorders (hallucination, delirium, visual hallucination)	7	3.27
Damage to the muscle and skeletal system (myalgia)	2	0.93

### 3.5 Evaluation of trigger efficiency

Analysis of 164 patient records without a positive trigger found 4 ADEs, yielding a 2.82% false-negative rate. Overall, 138 patients had both positive trigger detection and ADEs occurrence, 178 patients had positive trigger detection but no ADEs occurrence, four patients had ADEs occurrence without trigger detection, and 160 patients had neither trigger detection nor ADEs occurrence. A four-grid table was established to summarize the findings (refer to Table 6). The true positive rate (sensitivity) was calculated to be 97.2%, whereas the specificity was 47.3%, and the accuracy index was 0.45. The kappa value was measured to be 0.33.

**TABLE 6:** Relationship between trigger detection and WHO-UMC method determination.

Trigger check out	Determination of ADE by WHO-UMC evaluation method		Total
	Occurrence of ADE	No ADE occurred	
Positive	138	178	316
Negative	4	160	164
Total	142	338	480

WHO-UMC, World Health Organization-Uppsala Monitoring Center.

### 3.6 Risk factors associated with adverse events

Univariate analysis revealed that the length of hospital stay, the number of antifungal drugs used in combination, the number of other antibiotics used in combination, the number of positive triggers, and the number of comorbid diseases were identified as risk factors for ADEs (Table 3). The results from binary logistic regression indicated that a hospital stay between 11 and 20 days (OR = 1.904), 3 to 5 positive triggers (OR = 7.797), 6 or more positive triggers (OR = 27.975), and 4 to 6 comorbid diseases (OR = 2.669) were positively correlated with the occurrence of ADEs (*p* < 0.05) (Table 7).

**TABLE 7:** Binary logistic regression results of risk factors for the occurrence of ADEs.

Characteristics	OR (95% CI)	*p* value
Number of admission(days)		0.13
1–10	Reference	
1–20	1.90 (1.12∼3.23)	0.017*
21–30	2.16 (0.84∼5.60)	0.112
≥31	0.65 (0.24∼1.77)	0.401
Number of combined antifungal agents		0.146
0–1	Reference	
2	1.59 (0.84∼3.02)	0.154
3	3.20 (0.71∼14.46)	0.131
Number of combined antibacterials		0.959
0–3	Reference	
4–6	1.10 (0.51∼2.35)	0.811
≥7	0.92 (0.18∼4.63)	0.923
Number of positive triggers		<0.001*
0–2	Reference	
3–5	7.80 (4.43∼13.73)	<0.001*
≥6	27.98 (7.64∼102.51)	<0.001*
Number of combined diseases		0.002*
1–3	Reference	
4–6	2.67 (1.45∼4.91)	0.002*
>6	1.32 (0.67∼2.58)	0.425

OR, Odds ratio.

Bolded and asterisked results indicate that the data is less than 0.05, which is statistically significant.

## 4 Discussion

### 4.1 ADEs detection

In this study, a total of 21 trigger items were established based on the GTT method and refined through a literature search, trigger extraction and revision, and expert research. The effectiveness of these triggers in detecting ADEs was verified via retrospective analysis. The results revealed that the incidence rate of ADEs detected by the triggers was approximately 28.75%, which is consistent with the findings of de Souza et al. In contrast, the Spontaneous Reporting System (SRS) reported only 35 cases of ADEs during the same time period, with a voluntary reporting rate of 0.77% (33/4,282). The ADEs detection rate of the established triggers was 37 times greater than that of the hospital’s spontaneous reporting. Classen DC et al. conducted a study comparing three current ADEs monitoring methods in the United States, and their results demonstrated that the ADEs confirmed using the GTT method was at least ten times greater than that detected using other methods (Classen et al., 2011). Similarly, a study by SAJITH et al. confirmed the efficiency of the GTT method (Sajith et al., 2021). The findings of our study are consistent with these previous research findings, indicating that the triggers established in our study have greater sensitivity and can effectively supplement the voluntary reporting system, thus mitigating the problem of underreporting of ADEs in hospitals. Moreover, previous studies have focused primarily on specific populations or departments, with an emphasis on the influence of population factors on the research results. Unlike these studies, our research focused on antifungal drugs themselves and started by exploring the adverse reactions associated with these drugs. By combining relevant ADEs literature and data, we optimized the triggers using the Delphi method. The results of our study are similar to those obtained from population-based studies, making them valuable for the monitoring of ADEs caused by other drugs.

### 4.2 Validity of triggers

The PPV of the established triggers was 26.92%, with all 21 items triggering ADEs. Of these items, 20 were effective in detecting ADEs, 17 of which had a PPV greater than 15%. The most prevalent ADEs listed in the instructions for the use of antifungal drugs are gastrointestinal reactions and damage to liver and kidney function. Gastrointestinal reactions typically include mild symptoms such as nausea, vomiting, and diarrhea, which can be managed with symptomatic treatment or discontinuation of the drug. Damage to liver and kidney function is a significant factor that leads to the discontinuation of new drug research and clinical use. This type of damage can result in various degrees of harm, including hepatitis, cholestasis, fulminant liver failure, and renal failure (de Souza et al., 2016; Steimbach et al., 2017). In this study, the triggers for gastrointestinal reactions and liver and kidney function damage were identified as L4, L5, S1, S6, and S7, all of which had high PPVs. Additionally, trigger items such as L6, L7, and S9, which also had a high PPV and a high detection rate of ADEs, were primarily established based on the adverse reactions of the drugs themselves. For adverse reactions with an occurrence rate greater than 1%, it is possible to establish related trigger items to more accurately detect ADEs related to the drug in medical records. This approach can improve the overall detection rate of ADEs. Therefore, the formulation of trigger items based on the instructions for the drug is highly feasible.

Items with a lower PPV, such as S10, were determined to be frequent ADEs but had fewer trigger instances. However, due to the indications for phlebitis as a common and severe ADEs requiring prompt treatment in the instructions and relevant literature, phlebitis removal is currently not considered (Steimbach et al., 2017; Sajith et al., 2021).

Items with a higher PPV but fewer ADEs instances, such as L1 and S8, had positive frequencies of 30 and 9 instances, respectively. However, only 4 and 1 instances were ultimately determined to be ADEs. The potential causes of this discrepancy include the following: ① failure to exclude patients with hematological diseases, tumors, and critically ill patients who often have abnormal blood test results and are prone to false triggers; and ② the frequent appearance of the term “bleeding” in medical records, such as surgical patient records and transfusion protocols, resulting in a high false trigger rate for this term. In the future, it may be possible to exclude patients from certain specialized departments to improve the detection rate of ADEs for these trigger items.

Additionally, the number of trigger instances and ADEs determination instances for items T1 and T2 were both low. During the earlier Delphi method, experts did not propose any modifications to these items. This may be because intervention measures are considered emergency measures when ADEs occur. To maintain the integrity of the trigger items, these two items were not removed from this study and were further evaluated after expanding the retrospective sample.

### 4.3 Risk factors

The analysis of risk factors revealed that several factors contribute to ADEs caused by antifungal drugs. These factors included a hospital stay of 11–20 days, a positive trigger count of ≥3, a positive trigger count of ≥6, and 4–6 comorbid diseases.

The association between hospital stays and ADEs as a risk factor has long been debated. Tola WO et al. conducted a study on ADEs in cancer patients and reported that a hospital stay exceeding 8 days was associated with an increased risk of ADEs (Tola et al., 2023); Choi E et al. also supported this finding, stating that longer hospital stays increased the likelihood of ADEs occurrence (Choi et al., 2022). Consistent with previous studies, our study revealed that patients with a hospital stay of 11–20 days had a greater likelihood of experiencing ADEs. However, determining a causal association between ADEs and hospital stays is challenging because patients with longer hospital stays often require more medication or surgical treatment, providing them with more opportunities for ADEs to occur (Hwang et al., 2014). Further research is needed to confirm the causal association between extended hospital stays and ADEs.

Additionally, previous literature has identified the number of comorbid diseases as a risk factor for ADEs. Sendekie et al. (2023)’s study showed a strong link between ADE incidence and comorbidities. (*P* = 0.021) (*P* = 0.021). Angamo et al. (2017)’s study further supported this finding, indicating that having ≥4 comorbid diseases (AOR = 2.09; 95% CI = 1.27–3.44; *P* = 0.004) is an independent predictive factor for ADEs. Our study also revealed that having 4-6 comorbid diseases was a risk factor for ADEs caused by antifungal drugs, consistent with the results of Angamo MT et al. However, no significant difference was observed in groups with hospital stays exceeding 20 days or those with >6 comorbid diseases. Longer hospital stays and more comorbidities often indicate worse health, like severe metabolic issues or organ damage, potentially masking drug-related events and reducing identified ADEs, obscuring significant differences.

Hu et al. (2022) conducted a study using machine learning techniques to predict the occurrence of ADEs in elderly hospitalized patients. The findings revealed that patients in the ADEs group had an average of 3.2 trigger factors, whereas those in the non-ADEs group had only a 1.0 trigger factor. This suggests that patients with more trigger factors during hospitalization are more likely to experience ADEs, which aligns with our own findings. It is evident that the greater the number of trigger factors present, the greater the probability of ADEs in patients. This also indicates that the machine learning model utilized in this study performs well in identifying and quantifying ADEs in hospitalized patients.

These results collectively imply that when dealing with patients who have an extended hospital stay, multiple comorbidities, or who are using multiple antifungal drugs, medical staff should be actively involved in the treatment process. It is important to closely monitor every aspect of medication use, avoid incorrect combinations of drugs, and minimize the occurrence of ADEs as much as possible.

### 4.4 Research limitations

Although this study focused specifically on ADEs caused by antifungal drugs rather than on specific populations, it has several limitations. 1) First, this was a single-center trial with a limited case review period of only 2 years and a small sample size. Future research should aim to gather more accurate data by conducting studies with larger sample sizes and across multiple centers. 2) Second, this study is retrospective in nature, and the quality of medical record documentation can significantly impact the results, particularly in identifying trigger factors for clinical symptoms. Incomplete or omitted case records may affect the effectiveness of trigger detection for ADEs. 3) Finally, the triggers employed in this study were designed based on expected adverse events or known mechanisms of adverse events, thereby limiting their ability to identify and detect unexpected adverse events. In subsequent research, we aim to expand our retrospective record reviews to include more hospitals and continually refine the triggers used.

## 5 Conclusion

In summary, this retrospective analysis demonstrated that utilizing triggers based on the respective antifungal drug can effectively enhance the detection rate of ADEs. This approach can serve as a valuable tool for drug surveillance in medical institutions and offers guidance for the implementation of proactive monitoring research on ADEs. Furthermore, the integration of information technology, such as artificial intelligence and big data prediction models, can further enhance active drug monitoring systems. This advancement will enable the early, rapid, and intelligent identification of potential ADEs during medication use. Providing automatic prompts containing pertinent information to clinical medical staff and pharmacists enables them to make timely assessments and implement early preventive measures, ultimately enhancing the safety of medication administration.

## Data Availability

The raw data supporting the conclusion of this article will be made available by the authors, without undue reservation.

## Abbreviations

ADEs, Adverse Drug Events; ADR, Adverse Drug Reaction; AE, Adverse Event; GTT, Global Trigger Tool; PPV, Positive Predictive Value; IHI, Institute for Healthcare Improvement; WHO, World Health Organization; CHPS, China Hospital Pharmacovigilance system; HIS, Hospital Information System; CTCAE, Common Terminology Criteria for Adverse Events; UMC, Uppsala Monitoring Center.
